# Principal visionary leadership and teacher instructional innovation: mediating roles of teacher workplace well-being and collaboration

**DOI:** 10.3389/fpsyg.2026.1701099

**Published:** 2026-01-21

**Authors:** Chuang Yang, Zeqing Xu

**Affiliations:** 1 School of Education, Guangzhou University, Guangzhou, China; 2 Faculty of Education, East China Normal University, Shanghai, China

**Keywords:** instructional innovation, mediating effect, teacher collaboration, visionary leadership, workplace well-being

## Abstract

Instructional innovation is essential for preparing schools for the future and nurturing innovative talent. This study, grounded in vision communication theory, examines how principal visionary leadership contributes to teachers’ instructional innovation through psychological and organizational mechanisms. Survey data from 813 teachers in China were analyzed using structural equation modeling and bootstrapping. Results indicate that visionary leadership directly predicts instructional innovation and indirectly influences it through teacher workplace well-being and collaboration, with well-being exerting the strongest mediating effect. Furthermore, a chain pathway was identified: visionary leadership enhances teachers’ workplace well-being, which fosters greater collaboration and ultimately promotes innovation. These findings extend research on teacher innovation, provide cross-cultural evidence of visionary leadership’s effects, and suggest that school leaders can promote sustainable innovation by enhancing vision communication, cultivating a supportive school culture, and integrating teacher well-being with formal and informal collaboration structures.

## 1 Introduction

School systems are undergoing significant transformation driven by the integration of artificial intelligence and big data, which is reshaping teaching models, learning approaches, and assessment practices. This shift places growing demands on teachers’ capacity for innovation (Kim, 2024). In this context, instructional innovation, which is defined as the ongoing improvement and creative application of teaching beliefs, methods, content, and technologies, has become essential for preparing schools for the future, meeting diverse student needs, and nurturing innovative talent (Fullan, 2009; Da’as, 2023; Xiang et al., 2024). Consequently, identifying how to effectively foster and sustain teachers’ innovative practices has emerged as a critical focus in educational research.

Previous studies have shown that teacher innovation is influenced by multiple factors, including individual traits, workplace conditions, and leadership styles (Yang et al., 2025). Empirical evidence also suggests that principal leadership has an effect on teacher innovation (Liu and Hallinger, 2021; Vermeulen et al., 2022). However, systematic empirical studies focusing specifically on visionary leadership remain limited. Although vision communication has long been viewed as a core driver of effective leadership (Lewis and Clark, 2020), most empirical work has examined it within broad transformational leadership frameworks, where vision-related behaviors are bundled together with many other leadership dimensions. Such lumped approaches have been criticized for conceptual ambiguity and for obscuring the unique contribution of specific leader behaviors. Responding to calls for “split” conceptualizations of leadership (Carton, 2022), recent scholarship has begun to examine visionary leadership as a distinct construct, emphasizing leaders’ communication of a compelling future image to mobilize collective action. This shift provides an opportunity to more precisely understand the specific mechanisms through which visions influence followers.

In addition, the mechanisms linking leadership to teacher innovation are not yet fully understood (Polatcan et al., 2024). In Chinese schools, heavy instructional and administrative workloads, together with heightened emotional stress, make teachers’ well-being particularly important for sustaining innovation (Sun et al., 2025). Moreover, the strong tradition of collaboration, especially through Teacher Research Groups, underscores the critical role of teacher collaboration in fostering innovation (Haiyan and Allan, 2021). Building on these insights, this study incorporates teachers’ well-being and collaboration into a unified framework to examine how visionary leadership promotes instructional innovation.

Therefore, we address three questions: (1) Is principal visionary leadership significantly associated with teachers’ instructional innovation? (2) How does this association unfold within China’s basic education context? (3) What roles do teachers’ well-being and collaboration play in this process? Therefore, the study examines the impact of principal visionary leadership on teachers’ instructional innovation in China’s school context and is designed to make three contributions. First, by focusing on the visionary dimension of leadership, it refines evidence on how specific leadership types relate to teachers’ instructional innovation. Second, by integrating teachers’ well-being and collaboration into a single model, it identifies multiple mediating pathways through which leadership affects innovation. Third, using empirical data from Chinese schools, it offers context-sensitive evidence and practical implications for school leadership and teacher development strategies.

## 2 Literature review

### 2.1 Vision communication theory

Vision communication theory explains how leaders influence followers’ behavior by articulating and communicating a compelling image of a desired future (Kirkpatrick and Locke, 1996; Stam et al., 2014). Central to the theory is the idea that effective vision communication facilitates followers’ internalization of collective goals, enhances positive psychological states, and motivates concrete vision-pursuit behaviors aligned with organizational objectives (Van Knippenberg and Stam, 2014).

Applied to school contexts, principal visionary leadership may promote teachers’ instructional innovation through multiple pathways. First, clear and inspiring vision communication can directly motivate teachers to engage proactively in innovative teaching practices, particularly under conditions of change (Awamleh and Gardner, 1999; Griffin et al., 2010). Second, by strengthening teachers’ identification with school goals and enhancing their sense of professional meaning, visionary leadership can improve workplace well-being, which in turn supports experimentation and creative instructional efforts (Pozo-Rico et al., 2023). Third, when the communicated vision emphasizes collective goals and shared responsibility, teachers are more likely to engage in collaborative exchanges, facilitating knowledge sharing and joint problem solving (Liu and Sun, 2025).

**Figure 1:**
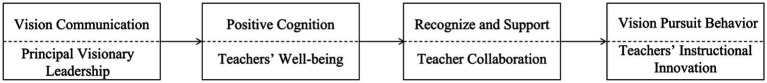
Framework based on the vision communication theory.

Importantly, vision communication theory also implies a sequential process: principals’ vision communication first enhances teachers’ well-being, which then fosters greater collaboration, ultimately translating into sustained instructional innovation as a form of vision pursuit behavior. Together, this perspective provides a concise theoretical foundation for the proposed chain mediation model (see Figure 1).

### 2.2 Visionary leadership and teacher instructional innovation

Research in educational leadership consistently shows that principal leadership largely shapes the direction of school change and teacher development (Daniëls et al., 2019). Visionary leadership can be understood as a leadership approach grounded in educational ideals and contextual insight. Its essence lies not in one-way declaration but in co-creation and shared ownership of a school vision that unites the community and drives continuous improvement (Aksu, 2009). According to Starratt (1995), visionary leadership involves five core elements: clarifying the vision (depicting the desired future state of the school), concretizing the vision (translating it into actionable goals for student learning and development), sharing the vision (engaging teachers, students, and parents in its construction), institutionalizing the vision (embedding it into the school’s mission, policies, and structures), and sustaining the vision (maintaining vitality through celebration and iterative renewal). Although visionary leadership is related to transformational leadership, it is conceptually distinct in emphasis and mechanism. Transformational leadership, as classically defined, comprises dimensions such as idealized influence, inspirational motivation, intellectual stimulation, and individualized consideration, and it broadly aims to raise followers’ motivation and performance (Bass and Avolio, 1994). Visionary leadership specifically foregrounds the content of an organizational future and the communicative processes by which leaders make that future meaningful to followers (Buss et al., 2023). Whereas transformational leadership often operates through developmental channels (e.g., stimulating problem-solving, providing individualized support), visionary leadership functions primarily through articulating a compelling picture of the future, aligning goals, and engaging followers’ affective commitment to shared objectives (Song et al., 2025; Dvir et al., 2004). Studies have shown that visionary leadership can enhance organizational support perceptions, strengthen employees’ self-efficacy, and promote affective commitment (Li et al., 2023; Du and Bao, 2023). At the organizational level, it has been linked to successful change implementation, higher performance, and improved stakeholder satisfaction (Mascareño et al., 2020). Despite these findings, scholars note that the literature on “pure” visionary leadership remains relatively limited, underscoring the need for further research on its mechanisms.

Teacher instructional innovation is generally defined as novel and useful attempts in areas such as curriculum design, teaching methods, classroom organization, and the application of educational technologies (Guskey, 1988). In recent years, research on teacher innovation has grown rapidly, with broad consensus on its positive effects on students’ learning satisfaction, academic self-efficacy, and achievement (Maun et al., 2023; Suyudi et al., 2022). However, teachers are facing heavy administrative workloads and emotional pressures nowadays. Some even exhibit a ‘lying flat’ attitude (Sun and Liao, 2025), characterized by low motivation and avoidance of additional innovative efforts. This phenomenon reflects a deeper loss of meaning in organizational goals. In such circumstances, principals’ visionary leadership becomes crucial. By articulating a clear and inspiring vision for education, principals can rekindle teachers’ sense of professional mission, restore their intrinsic motivation, and provide both value orientation and motivational support for instructional innovation. This study therefore focuses on visionary leadership to examine how its core mechanisms foster teachers’ innovative teaching practices.

Numerous empirical studies have shown that visionary leadership significantly enhances employee innovation. Cai et al. (2023) demonstrated that it fosters both individual and team-level innovation through goal-related mechanisms. In educational settings, principal visionary leadership offers clear strategic direction and robust organizational support, providing teachers with the resources and psychological safety needed for instructional experimentation (Zhu et al., 2019). Simultaneously, it nurtures shared values and a collective mission; by fostering trust and empowerment, it strengthens teacher autonomy and critical thinking (Makhrus et al., 2022), thereby encouraging the development and implementation of novel, effective teaching strategies. Chen and Yuan (2021) further found that such leadership stimulates creative teaching by enhancing teachers’ imaginative capacity.

Thus, we propose:

*H1*: Principal visionary leadership positively predicts teachers’ instructional innovation.

### 2.3 The mediating role of teacher workplace well-being

Teacher workplace well-being is generally defined as teachers’ overall positive evaluation of their professional life, encompassing both affective dimensions (e.g., positive/negative emotions) and cognitive dimensions (e.g., job satisfaction, sense of meaning) (Ozturk et al., 2024; Xia et al., 2025). A systematic review by Hascher and Waber (2021) concluded that subjective factors (e.g., self-efficacy, autonomy support, and social support) explain teacher well-being more strongly than objective demographic variables; Current interventions largely focus on generic psychological skills and rarely align with classroom realities. Therefore, we include teacher workplace well-being as a contextually relevant, malleable, and intervention-sensitive psychological variable in our framework, examining its role in the relationship between teachers’ perceived principal leadership and classroom instructional innovation.

Accumulating evidence supports this proposition. Lu et al. (2025), using survey data from 508 teachers, demonstrated that positive emotions broaden teachers’ cognitive resources, thereby enhancing perceived insider status and innovative behavior. Rahimi et al. (2024) similarly reported that teachers with higher quality of work life felt more psychologically empowered and adopted growth mindsets, making them more willing to try new teaching approaches. Research on Chinese EFL teachers (Qi and Derakhshan, 2025) further confirmed that positive emotions heighten classroom enthusiasm and enjoyment, which in turn stimulate instructional innovation.

Based on these findings, we propose:

*H2a*: Teachers’ workplace well-being is positively related to instructional innovation.

Principal leadership ranks as an essential factor for facilitating teacher workplace well-being. Empirical studies show that transformational, instructional, and authentic leadership enhance well-being directly and indirectly by supporting basic psychological needs and fostering positive work environments (Berkovich and Eyal, 2017; Abdulaziz Alfayez et al., 2024; Xu and Pang, 2024). Visionary leadership, in particular, centers on communicating a clear and compelling vision, may also positively affect teachers’ workplace well-being. By aligning goals, reducing uncertainty, and strengthening role identity and professional purpose, principals can improve teachers’ emotional experiences and overall workplace well-being (Bernards, 2023; Zhai et al., 2025). A shared vision is associated with higher organizational commitment and greater well-being (Zhu et al., 2011), and research confirms that school vision is central to teachers’ social and emotional well-being (Carroll et al., 2022).

Accordingly, we hypothesize:

*H2b*: Principal visionary leadership positively predicts teacher workplace well-being.

Integrating vision communication theory with the empirical evidence above, we propose a mediating pathway: principals who articulate a clear and inspiring vision enhance teachers’ identification with the school mission and their sense of professional meaning, thereby improving their workplace well-being. Elevated well-being, in turn, broadens teachers’ cognitive resources and strengthens their motivation to adopt new teaching approaches, ultimately translating into classroom and school-level instructional innovation. Research from Islam et al. (2024) also supports this pathway, showing that leaders promote innovation indirectly by enhancing employee well-being.

Therefore, we predict the following:

*H2*: Teachers’ workplace well-being mediates the relationship between principal visionary leadership and teachers’ instructional innovation.

### 2.4 The mediating role of teacher collaboration

Teacher collaboration refers to professional interactions and cooperative practices among teachers that are oriented toward instructional goals. These include joint lesson planning, classroom observation, peer feedback, co-design of teaching materials, and collective reflection. Unlike loose exchanges of information, collaboration emphasizes task interdependence and knowledge sharing centered on student learning. It also relies on trust, shared norms, and collective vision to sustain professional learning communities (PLCs) (Vescio et al., 2008). In Chinese schools, institutionalized structures such as teaching research groups, serve as the primary platforms for mutual support, knowledge dissemination, and the implementation of innovative practices (Wong, 2010).

Hence, we speculate that teacher collaboration is a critical organizational factor influencing instructional innovation. In China, where collectivism coexists with hierarchical structures, formal collaboration integrate dispersed individual knowledge into shared resources, reducing perceived resource barriers and risks associated with innovation (Chen, 2020; Gong, 2025). Recent empirical studies support this argument. Analyzing TALIS 2018 data from Taiwan, Pan et al. (2024) found that deeply engaged professional collaboration is significantly associated with school innovation and teachers’ innovative practices. Based on group dynamics and social cognitive theories, Qin et al. (2025) used structural equation modeling to show that collaboration enhances teaching motivation and efficacy, thereby promoting both innovative capacity and implementation.

We thus propose the following hypothesis:

*H3a*: Teacher collaboration positively predicts instructional innovation.

Visionary leadership enhances collaboration by articulating a compelling future and fostering shared commitment to collective goals (Van der Voet and Steijn, 2021). Principals’ consistent vision communication strengthens teachers’ emotional identification with the school’s mission, increasing their willingness to engage in knowledge sharing, coordinated efforts, and collaborative practice (Li et al., 2022). Wang’s (2018) qualitative study of two secondary schools in Northeast China suggested that principals’ visionary leadership played a central role in articulating a common vision, cultivating a culture of trust, and supporting collaborative practices, thereby driving continuous school improvement. Quantitative studies echo this, showing that school leaders’ vision is significantly and positively associated with both formal and informal teacher collaboration (Hsiao, 2022), and that principal support is a key condition for institutionalizing collaboration (Castro Silva et al., 2017).

Thus, we propose:

*H3b*: Principal visionary leadership is positively related to teacher collaboration.

According to vision communication theory and these findings, we outline the following pathway: through compelling vision communication, principals build shared goals and provide structural and psychological support for collaboration. Teachers, in turn, increase both the frequency and quality of collaborative practices, which facilitate the generation, experimentation, and diffusion of new ideas, ultimately advancing instructional innovation. Recent evidence also supports this mechanism; Teng et al. (2024) demonstrated that principal distributed leadership fosters teacher collaboration, which in turn enhances both team-level innovation willingness and individual innovation practice.

Based on the above analysis, the following hypothesis is proposed.

*H3*: Teacher collaboration plays a mediating role between principal visionary leadership and teachers’ instructional innovation.

### 2.5 The chain mediating role of teacher workplace well-being and collaboration

Positive emotions significantly promote altruistic and change-oriented organizational citizenship behaviors such as helping, offering suggestions, and proactive improvement (Chiaburu et al., 2022). These behaviors constitute the core mechanisms of teacher collaboration. In other words, teachers’ workplace well-being can, through resource-gain and social-exchange processes, be translated into positive attitudes and behaviors toward colleagues and the organization (Chen et al., 2022), thereby enhancing both the frequency and quality of collaborative practices. A growing body of research in education has also established that teacher well-being is positively associated with work engagement, collaborative orientation, and participation in professional learning communities (Dreer, 2023; Banerjee et al., 2017).

Accordingly, we propose:

*H4a*: Teacher workplace well-being positively predicts teacher collaboration.

With the lens of visionary communication theory, principal visionary leadership strengthens shared goals and directional clarity among staff. It also fosters a supportive and equitable school culture, enhancing teachers’ work-related well-being (Zhu et al., 2011) and promoting collaborative practices such as joint lesson planning and resource sharing (Meyer et al., 2023). These conditions collectively contribute to higher levels and greater sustainability of classroom instructional innovation.

Synthesizing the logic of H2b, H3a, and H4a, our last hypothesis is as follows.

*H4*: Principal visionary leadership positively predicts teachers’ instructional innovation through the chain mediating roles of teacher workplace well-being and teacher collaboration.

## 3 Research methodology

### 3.1 Data collection and participants

This study adopted a cluster random sampling strategy to recruit in-service teachers from 12 public primary and secondary schools in City S, China. City S was chosen because of its exemplary role in China’s basic education: the region is recognized for its high educational quality, balanced distribution of resources, and a teaching force that demonstrates strong awareness of and engagement in instructional innovation. These features provide an ideal setting to examine the influence of visionary leadership on teachers’ innovative practices.

Prior to data collection, the research team fully informed all participating teachers about the research purpose, participation procedures, and data usage. Participation was voluntary, and informed consent was obtained from all respondents. The confidentiality of individual responses was strictly maintained, and all data were used exclusively for academic purposes.

A total of 900 questionnaires were distributed, of which 813 valid responses were received, yielding a response rate of 90.33%. Among the respondents, 135 were male (16.61%) and 678 were female (83.39%). Regarding age, 257 teachers (31.61%) were 35 years old or younger, 319 (39.24%) were between 36 and 45 years old, and 237 (29.15%) were above 45. In terms of teaching experience, 68.39% had more than 10 years of experience. The majority of participants held a bachelor’s degree (85.24%). In addition, 159 respondents (19.56%) concurrently served in middle-level leadership roles such as research group leaders.

### 3.2 Measures

Before describing each construct, it is important to note that all measures used in this study were adapted from well-established and widely validated scales. Given the educational context of the present research, we conducted minor wording adjustments to ensure contextual relevance while maintaining the original constructs’ conceptual integrity. A standard translation and back-translation procedure (Brislin, 1980) was employed. No items were added or removed, as all scale items demonstrated clear meaning, theoretical consistency, and satisfactory factor loadings. Each scale has been extensively applied in empirical studies in education, supporting their suitability for teacher samples.

Following Fornell and Larcker (1981), Cronbach’s *α* and composite reliability (CR) values above 0.70 were considered indicative of acceptable internal consistency. Regarding structural validity, we adopted commonly used criteria in SEM research (Wen et al., 2004). Specifically, χ^2^/df values below 10, RMSEA and SRMR values below 0.10, and incremental fit indices (NFI and TLI) above 0.90 were regarded as indicating acceptable model fit. Although RMSEA values below 0.08 are often recommended as a stricter benchmark, previous methodological studies have noted that RMSEA is sensitive to model complexity and sample size, and values below 0.10 may still indicate an acceptable fit when considered jointly with other fit indices (Wen et al., 2004). Detailed information regarding scale origins, sample items, reliability, validity evidence, and CFA results is presented below.

#### 3.2.1 Visionary leadership

This construct was measured using the Visionary Inspiration subscale of the transformational leadership scale developed by Li and Shi (2005). Van Knippenberg and Sitkin (2013) argued that visionary leadership can be studied independently from charismatic–transformational leadership, and this scale has been validated in educational contexts with satisfactory reliability and validity. This subscale specifically captures leaders’ behaviors in articulating and communicating a compelling vision to guide collective action, which aligns closely with the narrower construct of visionary leadership (Van Knippenberg and Stam, 2014). The six items reflect key aspects of vision communication in educational settings, such as clarifying the school’s philosophy and development goals and explaining the long-term significance of teachers’ work. Responses were rated on a five-point Likert scale (1 = strongly disagree, 5 = strongly agree), with higher scores indicating stronger perceptions of visionary leadership. In this study, Cronbach’s *α* was 0.969. Confirmatory factor analysis (CFA) indicated good model fit (χ^2^/df = 6.025, RMSEA = 0.079, NFI = 0.994, TLI = 0.989).

#### 3.2.2 Workplace well-being

We measured teachers’ workplace well-being using the six-item scale developed by Zheng et al. (2015), originally designed in the Chinese corporate context and widely applied in empirical studies of teacher well-being (Li et al., 2024). Sample items include: ‘I am basically satisfied with the specific content of my work’ and ‘I am basically satisfied with the sense of achievement I gain from my current job.’ A seven-point Likert scale was used (1 = strongly disagree, 7 = strongly agree). Higher scores reflected higher levels of workplace well-being. Cronbach’s *α* in this study was 0.941, and CFA results showed good fit (χ^2^/df = 8.350, RMSEA = 0.095, NFI = 0.985, TLI = 0.975).

#### 3.2.3 Teacher collaboration

Teacher collaboration was assessed using the five-item Collaborative Activity scale developed by Leithwood et al. (2006), which has been validated in Chinese schools (Zheng et al., 2020). Example items include: ‘During lesson preparation, I often receive useful suggestions from colleagues’ and ‘I make a conscious effort to coordinate the content of my courses with other teachers.’ All items were rated on a five-point Likert scale ranging from 1 (strongly disagree) to 5 (strongly agree), with higher scores reflecting stronger teacher collaboration. The Cronbach’s α coefficient was 0.920, with CFA results supporting good fit (χ^2^/df = 4.614, RMSEA = 0.067, NFI = 0.992, TLI = 0.988).

#### 3.2.4 Instructional innovation

We adopted a five-item scale developed by Liu et al. (2016) in educational settings to measure teachers’ instructional innovation. Sample items include: ‘I apply new methods to solve teaching problems’ and ‘I try out new teaching methods in my lessons.’ Responses were recorded on a five-point Likert scale (1 = strongly disagree, 5 = strongly agree). Higher scores reflected greater levels of teachers’ instructional innovation. In this study, Cronbach’s α was 0.958, and CFA results confirmed good fit (χ^2^/df = 3.026, RMSEA = 0.050, NFI = 0.998, TLI = 0.996).

#### 3.2.5 Control variables

Prior research has shown that demographic characteristics can influence teachers’ innovative teaching practices. Considering the existing literature (Zhu and Engels, 2014; Cai and Tang, 2021), the study controlled for gender, age, teaching experience, education, position, and homeroom teacher status.

### 3.3 Preliminary analysis

Common method bias (CMB) is a potential concern when the data are collected via self-reported questionnaires. To mitigate this risk, several procedural remedies were employed, including the use of reverse-coded items and assurances of confidentiality. Statistically, Harman’s single-factor test was conducted. The first unrotated factor accounted for 39.646% of the total variance, which is below the 50% threshold, indicating that CMB was not a serious issue (Podsakoff et al., 2003).

In addition, confirmatory factor analysis (CFA) was conducted to assess the discriminant validity of the four latent constructs in this study: visionary leadership (VL), teachers’ workplace well-being (TWB), teacher collaboration (TC), and teachers’ instructional innovation (TII). To evaluate model fit, several competing models were compared (Table 1). The hypothesized four-factor model (M1), in which each construct was treated as distinct, demonstrated superior fit indices (χ^2^/df = 7.667, NFI = 0.925, TLI = 0.925, RMR = 0.022) relative to alternative models that combined constructs (M2: VL + TWB; TC; TII; M3: VL + TWB + TC; TII; M4: VL + TWB + TC + TII), indicating that collapsing factors significantly worsened model fit. These results provide strong evidence for the discriminant validity of the four constructs and support their treatment as conceptually distinct variables in subsequent structural modeling.

**Table 1:** Comparison of measurement models.

Model	χ^2^/*df*	RMR	NFI	TLI
M1 (Hypothesized model): VL; TWB; TC; TII	7.667	0.022	0.925	0.925
M2: VL + TWB; TC; TII	25.615	0.153	0.744	0.721
M3: VL + TWB + TC; TII	34.377	0.149	0.654	0.622
M4: VL + TWB + TC + TII	52.796	0.148	0.466	0.414

VL, Visionary leadership; TWB, Teachers’ workplace well-being; TC, Teacher collaboration; TII, Teachers’ instructional innovation.

## 4 Results

### 4.1 Descriptive statistics

Descriptive statistics and Pearson correlations among the four main variables were conducted using SPSS 26.0. Table 2 presents the means (M), standard deviations (SD), and correlation coefficients. Principal visionary leadership was positively correlated with teacher workplace well-being (*γ* = 0.420, *p* < 0.01), teacher collaboration (γ = 0.593, *p* < 0.01), and instructional innovation (γ = 0.441, *p* < 0.01). Teacher workplace well-being was positively correlated with teacher collaboration (γ = 0.467, *p* < 0.01) and instructional innovation (γ = 0.534, *p* < 0.01). In addition, teacher collaboration showed a significant positive correlation with instructional innovation (γ = 0.464, *p* < 0.01). These results are consistent with the study’s expectations and provide preliminary support for subsequent hypothesis testing.

**Table 2:** Descriptive and correlation analysis (*N* = 813).

Variable	M	SD	1	2	3	4
1. VL	4.641	0.563	-			
2. TC	4.527	0.566	0.593**	-		
3. TII	4.155	0.722	0.441**	0.464**	-	
4. TWB	5.828	0.874	0.420**	0.467**	0.534**	-

M, mean; SD, standard deviation. *p < 0.05; **p < 0.01.

### 4.2 Testing the main effect

Structural equation modeling (SEM) was conducted to examine the relationships among the variables, with visionary leadership as the antecedent, instructional innovation as the outcome, and teacher workplace well-being and collaboration as mediators. The overall model fit was acceptable (χ^2^/df = 7.667, CFI = 0.934, NNFI = 0.925, SRMR = 0.037), allowing for further interpretation.

**Figure 2:**
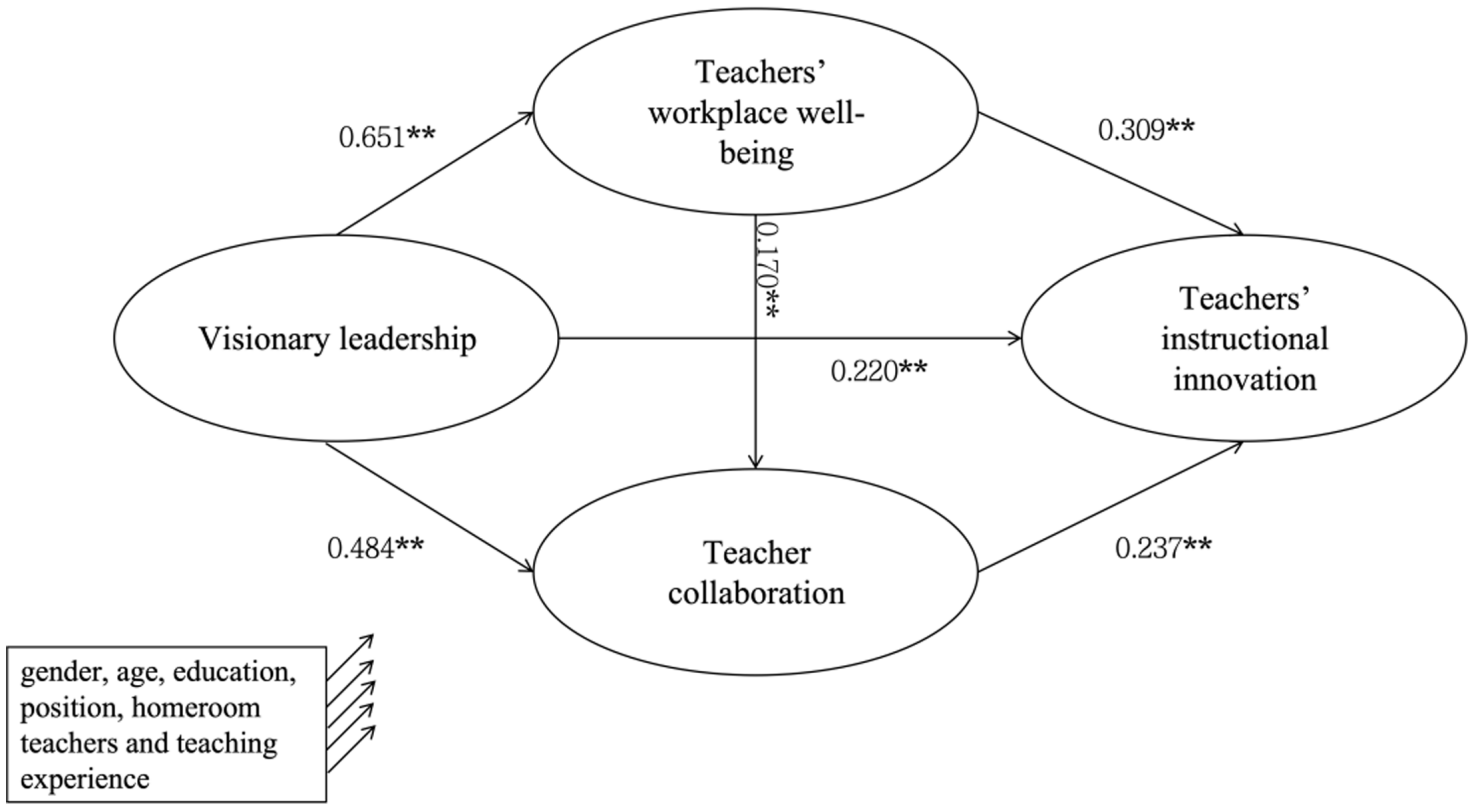
Structural equation modeling of visionary leadership and teachers’ instructional innovation.

The SEM results (see Figure 2) indicated that visionary leadership significantly predicted instructional innovation (*β* = 0.220, *p* < 0.01), supporting H1. This represents a small-to-moderate effect, suggesting that while visionary leadership contributes meaningfully to innovation, other factors also play a role. Visionary leadership also exerted significant positive effects on teacher workplace well-being (*β* = 0.651, *p* < 0.01) and teacher collaboration (*β* = 0.484, *p* < 0.01), supporting H2b and H3b. Furthermore, teacher workplace well-being significantly predicted both instructional innovation (*β* = 0.309, *p* < 0.01) and teacher collaboration (*β* = 0.170, *p* < 0.01), supporting H2a and H4a. Teacher collaboration also positively predicted instructional innovation (*β* = 0.237, *p* < 0.01), supporting H3a.

### 4.3 Testing the mediated effects model

Consistent with Preacher et al. (2007), we tested mediating effects using a bootstrapping procedure implemented via an SPSS macro. This non-parametric approach estimates indirect effects through repeated resampling, making no assumptions about the normality of the sampling distribution and thus offering robustness with non-normally distributed data. We estimated 95% bias-corrected CIs for indirect effects with 5000 bootstrapped re-samples.

Results of the mediation tests are presented in Table 3. The overall indirect effect of visionary leadership on instructional innovation was significant, with the 95% confidence interval excluding zero. Three specific mediation pathways were identified:

**Table 3:** Test of mediating effect.

Effect type		Effect	LLCI	ULCI	Two-tailed Sig(p)	Ratio
Direct effect: VL → TII		0.220	0.130	0.309	0.000	39.146%
Indirect effects	Path 1: VL → TWB → TII	0.201	0.113	0.203	0.000	35.765%
	Path 2: VL → TC → TII	0.115	0.052	0.131	0.000	20.463%
	Path 3: VL → TWB → TC → TII	0.026	0.010	0.034	0.000	4.626%
Total effect		0.562	0.482	0.641	0.000	100.00%

LLCI refers to the lower limit of the 95% estimated value interval, and ULCI refers to the upper limit of the 95% estimated value interval.

Path 1: Visionary leadership → Teacher workplace well-being → Instructional innovation. The indirect effect was 0.201, with a 95% CI of [0.113, 0.203]. This pathway accounted for 35.765% of the total effect, indicating that teacher workplace well-being partially mediated the relationship between principal visionary leadership and teachers’ instructional innovation. Thus, H2 was supported.

Path 2: Visionary leadership → Teacher collaboration → Instructional innovation. The indirect effect was 0.115, with a 95% CI of [0.052, 0.131], explaining 20.463% of the total effect. This finding indicates that teacher collaboration significantly mediated the link between principal visionary leadership and teachers’ instructional innovation, supporting H3.

Path 3: Visionary leadership → Teacher workplace well-being → Teacher collaboration → Instructional innovation. The indirect effect was 0.026, with a 95% CI of [0.010, 0.034], accounting for 4.626% of the total effect. This result confirms the significance of the chain mediation of teachers’ workplace well-being and collaboration, supporting H4.

## 5 Discussion

Grounded in vision communication theory, this study constructed a chain mediation model to examine the mechanisms linking principal visionary leadership with teachers’ instructional innovation. Drawing on data from Chinese schools, we contribute to the growing international understanding of leadership’s pivotal role in engaging teachers in school improvement (Murphy, 2013). Our findings respond to recent calls for cross-cultural evidence on leadership effectiveness (Zhang et al., 2021) and offer practical insights into building innovative teacher communities. In this sections, we will further interpret the results, propose managerial implications, and outline directions for future research.

### 5.1 Interpretation of the findings

#### 5.1.1 The direct relationship between visionary leadership and instructional innovation

Our study revealed a significant positive relationship between principal visionary leadership and teachers’ instructional innovation, persisting after controlling for demographic variables. Principals who articulate a clear, shared, and institutionalized vision directly enhance teachers’ willingness and frequency in adopting innovative teaching practices. Specifically, such leadership is associated with teachers’ efforts to apply new pedagogical approaches, experiment with engaging instructional methods, and integrate technology to improve learning outcomes.

This finding aligns with a growing body of research in organizational behavior, which demonstrates that visionary leadership fosters creativity at multiple levels—by strengthening team goal commitment at the collective level and by aligning leader–follower goals at the individual level (Cai et al., 2023). Extending these insights into the context of Chinese basic education, the present study provides direct empirical evidence that visionary leadership can translate into classroom-level instructional innovation, thus addressing a gap in the educational leadership literature.

Furthermore, our results resonate with the accumulating evidence on the positive role of supportive leadership styles in stimulating teacher innovation, such as the demonstrated impact of transformational leadership on innovative teaching practices (Kılınç et al., 2024). However, what distinguishes the present study is its focus on the vision-oriented dimension of leadership. Prior work often bundles vision with other charismatic behaviors, this conflation obscures its unique effects. The findings suggest that the clarity and shared nature of a school’s vision possess a unique motivational force that can directly drive teacher innovation, without necessarily relying on the broader set of transformational leadership behaviors. In other words, visionary leadership represents not just a subset of transformational leadership but a distinct mechanism that emphasizes goal direction and meaning-making as critical levers for instructional innovation.

#### 5.1.2 The independent mediating roles of teacher workplace well-being and collaboration

We identified a dual pathway through which principal visionary leadership is associated with instructional innovation. Teacher workplace well-being and teacher collaboration serve as independent mediators, indicating that visionary leadership promotes innovation both directly and through distinct psychological and organizational mechanisms.

Notably, among all mediating pathways, teacher workplace well-being emerged as the most influential, accounting for 35.77% of the total effect. It is substantially more than collaboration (20.46%) or the sequential chain (4.63%). One plausible explanation lies in the affective primacy of well-being: visionary leadership articulates a compelling future that fulfills teachers’ core psychological needs for meaning, competence, and belonging. When teachers experience higher well-being, they are not only more resilient but also more cognitively flexible and intrinsically motivated to experiment with novel instructional approaches. This novel result directly responds to the research gap identified by Thurlings et al. (2015), who emphasized that the psychological mechanisms of teacher innovation remain a largely unexplored ‘black box’. By empirically validating the mediating role of well-being, this study demonstrates that a shared vision can simultaneously enhance teachers’ sense of well-being and organizational commitment, which in turn translates into classroom improvements. In doing so, it offers new avenues for investigating visionary leadership from emotional and psychological perspectives (Zhu et al., 2011). This contribution also extends the scope of teacher well-being research. As noted in Zhang et al.’s (2024) systematic review, the majority of studies have focused on antecedents of teacher well-being (such as individual, organizational, and contextual factors), whereas only 18.22% examined its outcomes. By highlighting teacher well-being not merely as a consequence of leadership style but also as a crucial psychological antecedent of innovation, this study broadens the conceptual boundaries of well-being research.

Teacher collaboration was also shown to play a significant mediating role (indirect effect = 0.115, 95% CI [0.052, 0.131]), explaining about 20.5% of the total effect. Collaboration facilitates the translation of ideas into classroom practices through mechanisms such as knowledge sharing, peer feedback, and collective reflection (Vescio et al., 2008). Moreover, collaboration is strongly influenced by school climate and leadership style (Ma and Marion, 2024). In collectivist cultural contexts, this mechanism becomes particularly salient: school leaders can more effectively mobilize teachers to work together, thereby alleviating individual workload and enhancing collective willingness to innovate through shared wisdom (Teng et al., 2024). Drawing on survey data from 813 teachers across Chinese primary and secondary schools, the present study provides robust evidence for this logic, offering further insights into how principals leverage collaboration to support and sustain instructional innovation. This finding also resonate with research beyond education. For instance, van der Voet and Steijn (2021) demonstrated in a longitudinal study of Dutch social welfare teams that visionary leadership enhances team cohesion, which in turn fosters team innovation. By confirming the applicability of this dynamic in the school context, the current study illustrates how principal visionary leadership cultivates a collaborative climate that creates opportunities for collective exploration and experimentation, ultimately driving sustained instructional innovation.

#### 5.1.3 The chain mediating role of teacher workplace well-being and collaboration

We further identifies a significant chain mediating mechanism linking principal visionary leadership to teachers’ instructional innovation, in which teacher workplace well-being enhances collaboration among colleagues, ultimately translating into innovative practices in the classroom. Though this sequential path accounted for only 4.6% of the total effect (indirect effect = 0.026, 95% CI [0.010, 0.034]), its statistical significance affirms the chain mediating mechanism in driving change. Visionary leadership not only establishes cognitive alignment with shared goals but also fosters teacher well-being, increasing openness and capacity for collaboration. In turn, a collaborative climate provides opportunities for resource sharing, peer support for experimentation, and collective learning, thereby fostering instructional innovation.

Using a chain mediation framework, our study advances vision communication theory by specifying the mechanisms through which principal visionary leadership translates into teachers’ instructional innovation. While prior research has primarily highlighted the motivational and goal-alignment functions of visionary leadership (Carton et al., 2014), less attention has been given to the affective and relational processes that convert a leader’s vision into concrete innovative practices (Shamir et al., 1993; Chen and Shi, 2024). Our findings show that teachers’ workplace well-being acts as a crucial affective link, facilitating the internalization of the school vision, which in turn fosters collaboration and ultimately promotes innovation. By explicitly modeling this affective–relational chain, the study not only clarifies how vision communication operates in practice but also extends theory beyond prior models that primarily focused on direct motivational effects.

In addition, the findings respond to Liu et al.’s (2024) critique that research on teacher innovation often suffers from weak theoretical grounding. By integrating vision communication theory into a chain mediation model and subjecting it to empirical validation, this study advances a more theory-driven account of teacher innovation. In doing so, it contributes to a shift from predominantly descriptive or correlational approaches toward explanatory, mechanism-based research (Thurlings et al., 2015).

### 5.2 Practical implications

First, enhancing principals’ visionary leadership is crucial for fostering teachers’ instructional innovation. Under the principal responsibility system that prevails in Chinese schools, principals play a decisive role in shaping the direction of school development and mobilizing teachers (Zhang et al., 2025). The findings of this study suggest that principals should continuously strengthen their capacity for visionary leadership. This requires not only the formulation of clear, inspiring, and feasible visions, but also their effective communication to teachers. Communication should occur through both formal channels (e.g., staff meetings and strategic planning sessions), and informal, subtle interactions (e.g., everyday conversations and expressions of care). Moreover, principal training and selection should place greater emphasis on vision communication, emotional intelligence, and teacher motivation, equipping school leaders to transform abstract visions into shared commitments and concrete actions. This echoes earlier work highlighting that the effectiveness of a vision depends not merely on its content but also on how leaders convey and enact it in daily practice (Carton et al., 2014).

Second, cultivating teacher well-being should become a strategic priority in school management. Our study underscores that teacher workplace well-being extends beyond individual job satisfaction; it is also a driving force for collaboration and instructional innovation. Accordingly, school leaders should incorporate teacher well-being into long-term planning. Concrete measures might include balancing workloads, providing sustained opportunities for professional growth, and establishing recognition and reward systems that value both teaching and innovation (Aelterman et al., 2007). Empirical research further shows that well-being is shaped by teachers’ goal orientations and coping strategies when facing challenges (Parker et al., 2012). Principals can foster mastery-oriented coping by introducing initiatives such as ‘failure-sharing sessions’ or ‘micro-goal journals’, thereby helping teachers reframe setbacks as opportunities for growth. In this way, schools can build a supportive culture that sustains and enhances teacher well-being over time.

Third, strengthening teacher collaboration provides a practical pathway to innovation. While many Chinese schools have institutionalized collaboration through teaching and research groups, curriculum development teams, and peer observations, informal collaboration remains equally important. Spontaneous exchanges in the office, mentoring conversations, and online peer support networks also play vital roles. There is an imperative need for school leaders to create favorable conditions for collaboration through organizational design and resource support, such as allocating time for joint lesson planning, recognizing collaborative achievements, and encouraging cross-disciplinary exchange to break down subject silos. Such efforts can nurture whole-school professional learning communities (Haiyan and Allan, 2021). Systematic reviews further demonstrate that a strong culture of collaboration enhances teacher efficacy, collective learning, and instructional innovation (Vangrieken et al., 2015). Thus, innovation cannot rely solely on principals’ vision or individual teachers’ efforts. Instead, it must be embedded in both formal and informal networks of collaboration, where teacher well-being, professional relationships, and collective creativity are interwoven into a sustainable culture of school-wide innovation.

### 5.3 Limitations and future research

While this study offers meaningful theoretical and practical insights, several limitations should be acknowledged, and these also suggest promising directions for future research.

First, the study adopted a cross-sectional design, which allows us to examine statistical associations and mediated pathways, but does not permit strong causal inferences or capture developmental processes over time. Consequently, the mechanisms identified in this study should be interpreted as correlational rather than causal. Future research could employ longitudinal, experimental, or experience-sampling designs to more rigorously test the temporal ordering and potential causal pathways linking principals’ visionary leadership with teacher well-being, collaboration, and instructional innovation.

Second, the sample was drawn from a single region in China. As regional educational policies, leadership norms, and school resources may shape teachers’ perceptions and behaviors, the generalizability of the findings may be limited. Future studies could expand sampling to multiple provinces or conduct cross-national comparisons to assess the extent to which the observed relationships hold across different educational systems and cultural contexts.

Third, all focal variables were measured through teacher self-reports, which may introduce common method variance or social desirability bias. Although this approach is widely used in leadership and organizational behavior research, future studies could strengthen the validity of findings by incorporating multiple data sources, such as principal logs, peer evaluations, student reports, or observational data. Mixed-methods designs would also provide richer and more fine-grained insights into how visionary leadership is enacted in practice and how it relates to teachers’ psychological states and innovative behaviors.

Finally, while this study provides empirical evidence for the pathways through which visionary leadership relates to teachers’ instructional innovation, it is important to acknowledge that these relationships may not be uniform across all school contexts. A growing body of research highlights the importance of considering contextual moderators when examining leadership effects (Hallinger, 2018), suggesting that the enactment and impact of visionary practices may vary depending on specific school conditions. For instance, school size may influence leaders’ ability to communicate a cohesive vision and sustain close teacher interactions; in smaller schools, relational proximity may strengthen the effects of visionary leadership on well-being and collaboration, whereas in larger schools, hierarchical layers may dilute these influences. Future research could incorporate these contextual features as moderators or adopt multi-level comparative designs to better understand when and where visionary leadership most effectively promotes teacher innovation.

## 6 Conclusion

This study constructed and tested a chained mediation model to examine how principal visionary leadership may relate to teachers’ instructional innovation through the dual pathways of teacher workplace well-being and collaboration. Drawing on survey data from 813 primary and secondary school teachers in China, the findings demonstrate that visionary leadership exerts both direct and indirect effects on instructional innovation. Specifically, teacher well-being and collaboration function as independent mediators, and more importantly, they form a chain mediation pathway that links visionary leadership to instructional innovation.

## Data Availability

The raw data supporting the conclusions of this article will be made available by the authors, without undue reservation.

## References

[ref1] Abdulaziz Alfayez A. Noman M. Saeed Alqahtani A. Ibrahim Altuwaijri A. Kaur A. (2024). Principal’s learning-centred leadership practices and teacher’s wellbeing: a self-determination theory perspective. Educ. Stud. 50, 448–466. doi: 10.1080/03055698.2021.1960150

[ref2] Aelterman A. Engels N. Van Petegem K. Pierre Verhaeghe J. (2007). The well-being of teachers in Flanders: the importance of a supportive school culture. Educ. Stud. 33, 285–297. doi: 10.1080/03055690701423085

[ref3] Aksu A. (2009). Total quality management and visionary leadership in primary schools. Egitim Bilim 34:99.

[ref4] Awamleh R. Gardner W. L. (1999). Perceptions of leader charisma and effectiveness: the effects of vision content, delivery, and organizational performance. Leadersh. Q. 10, 345–373. doi: 10.1016/S1048-9843(99)00022-3

[ref5] Banerjee N. Stearns E. Moller S. Mickelson R. A. (2017). Teacher job satisfaction and student achievement: the roles of teacher professional community and teacher collaboration in schools. Am. J. Educ. 123:932. doi: 10.1086/689932

[ref6] Bass B. M. Avolio B. J. (1994). Improving organizational effectiveness through transformational leadership. Thousand Oaks, CA: Sage.

[ref7] Berkovich I. Eyal O. (2017). The mediating role of principals’ transformational leadership behaviors in promoting teachers’ emotional wellness at work: a study in Israeli primary schools. Educ. Manag. Adm. Leadersh. 45, 316–335. doi: 10.1177/1741143215617947

[ref8] Bernards B. (2023). Do visionary and servant leaders reduce cognitive uncertainty of professionals? A study of team-based settings in public organizations. Public Manag. Rev. 25, 1059–1081. doi: 10.1080/14719037.2021.2005326

[ref9] Brislin R. W. (1980). Translation and content analysis of oral and written materials. Methodology 2, 389–444.

[ref10] Buss M. Kearney E. Noureen R. Gandhi N. (2023). Antecedents and effects of visionary leadership: when and how leader work centrality is linked to visionary leadership and follower turnover intentions. J. Leadersh. Organ. Stud. 30, 413–427. doi: 10.1177/15480518231203637

[ref11] Cai W. Fan X. Wang Q. (2023). Linking visionary leadership to creativity at multiple levels: the role of goal-related processes. J. Bus. Res. 167:114182. doi: 10.1016/j.jbusres.2023.114182

[ref12] Cai Y. Tang R. (2021). School support for teacher innovation: mediating effects of teacher self-efficacy and moderating effects of trust. Think. Skills Creat. 41:100854. doi: 10.1016/j.tsc.2021.100854

[ref13] Carroll A. Bower J. M. Chen H. Watterston J. Ferguson A. (2022). Schoolwide approaches for promoting social and emotional well-being in australian school contexts: focus group interviews with system and school stakeholders. Am. J. Educ. 129, 109–138. doi: 10.1086/721798

[ref14] Carton A. M. (2022). The science of leadership: a theoretical model and research agenda. Annu. Rev. Organ. Psychol. Organ. Behav. 9, 61–93. doi: 10.1146/annurev-orgpsych-012420-091227

[ref15] Carton A. M. Murphy C. Clark J. R. (2014). A (blurry) vision of the future: how leader rhetoric about ultimate goals influences performance. Acad. Manag. J. 57, 1544–1570. doi: 10.5465/amj.2012.0101

[ref16] Castro Silva J. Amante L. Morgado J. (2017). School climate, principal support and collaboration among Portuguese teachers. Eur. J. Teach. Educ. 40, 505–520. doi: 10.1080/02619768.2017.1295445

[ref17] Chen L. (2020). A historical review of professional learning communities in China (1949-2019): some implications for collaborative teacher professional development. Asia Pac. J. Educ. 40, 373–385. doi: 10.1080/02188791.2020.1717439

[ref18] Chen S. Luo Y. Mai Z. Chen X. Shen T. (2022). The mediating effect of subjective well-being in the relationship between social support and professional commitment among mainland Chinese kindergarten teachers. Front. Psychol. 13:1011855. doi: 10.3389/fpsyg.2022.1011855, 36237667 PMC9551680

[ref19] Chen R. Shi Y. J. (2024). “Before Merlin, the three armies pursue”: a study on the influence of visionary leadership on subordinates’ vision support behavior. Human Resourc. Dev. China 41, 82–93. doi: 10.16471/j.cnki.11-2822/c.2024.6.006

[ref20] Chen H. H. Yuan Y. H. (2021). The study of the relationships of teacher’s creative teaching, imagination, and principal’s visionary leadership. SAGE Open 11:21582440211029932. doi: 10.1177/21582440211029932

[ref21] Chiaburu D. S. Oh I. S. Stoverink A. C. Park H. H. Bradley C. Barros-Rivera B. A. (2022). Happy to help, happy to change? A meta-analysis of major predictors of affiliative and change-oriented organizational citizenship behaviors. J. Vocat. Behav. 132:103664. doi: 10.1016/j.jvb.2021.103664

[ref22] Da’as R. A. (2023). Teacher’s engagement in creativity: the role of school middle leaders’ values, team diversity and team knowledge self-efficacy. Think. Skills Creat. 49:101346. doi: 10.1016/j.tsc.2023.101346

[ref23] Daniëls E. Hondeghem A. Dochy F. (2019). A review on leadership and leadership development in educational settings. Educ. Res. Rev. 27, 110–125. doi: 10.1016/j.edurev.2019.02.003

[ref24] Dreer B. (2023). On the outcomes of teacher wellbeing: a systematic review of research. Front. Psychol. 14:1205179. doi: 10.3389/fpsyg.2023.1205179, 37575417 PMC10421665

[ref25] Du M. Bao Z. (2023). Visionary leadership and employee voice behavior: mediating role of self-efficacy. Soc. Behav. Pers. 51, 68–74. doi: 10.2224/sbp.12228

[ref26] Dvir T. Kass N. Shamir B. (2004). The emotional bond: vision and organizational commitment among high-tech employees. J. Organ. Chang. Manag. 17, 126–143. doi: 10.1108/09534810410530575

[ref27] Fornell C. Larcker D. F. (1981). Evaluating structural equation models with unobservable variables and measurement error. J. Mark. Res. 18, 39–50. doi: 10.2307/3151312

[ref28] Fullan M. (2009). Large-scale reform comes of age. J. Educ. Change 10, 101–113. doi: 10.1007/s10833-009-9108-z

[ref29] Gong J. (2025). The effects of distributed leadership on teaching innovation in Shanghai, China: the mediating roles of teacher autonomy, teacher collaboration, and teacher self-efficacy. Front. Psychol. 16:1562838. doi: 10.3389/fpsyg.2025.1562838 40727058 PMC12301307

[ref30] Griffin M. A. Parker S. K. Mason C. M. (2010). Leader vision and the development of adaptive and proactive performance: a longitudinal study. J. Appl. Psychol. 95, 174–182. doi: 10.1037/a0017263, 20085414

[ref31] Guskey T. R. (1988). Teacher efficacy, self-concept, and attitudes toward the implementation of instructional innovation. Teach. Teach. Educ. 4, 63–69. doi: 10.1016/0742-051x(88)90025-x

[ref32] Haiyan Q. Allan W. (2021). Creating conditions for professional learning communities (PLCs) in schools in China: the role of school principals. Prof. Dev. Educ. 47, 586–598. doi: 10.1080/19415257.2020.1770839

[ref33] Hallinger P. (2018). Bringing context out of the shadows of leadership. Educ. Manage. Adm. Leadersh. 46, 5–24. doi: 10.1177/1741143216670652

[ref34] Hascher T. Waber J. (2021). Teacher well-being: a systematic review of the research literature from the year 2000–2019. Educ. Res. Rev. 34:100411. doi: 10.1016/j.edurev.2021.100411

[ref35] Hsiao C. C. (2022). Effects of creative self-efficacy and work value on creative teaching: a crosslevel analysis of professional learning communities and teachers’ trust. J. Res. Educ. Sci. 67, 255–289.

[ref37] Islam T. Zulfiqar I. Aftab H. Alkharabsheh O. H. M. Shahid M. K. (2024). Testing the waters! The role of ethical leadership towards innovative work behavior through psychosocial well-being and perceived organizational support. J. Organ. Change Manag. 37, 1051–1072. doi: 10.1108/jocm-09-2023-0382

[ref38] Kılınç A. Ç. Polatcan M. Savaş G. Er E. (2024). How transformational leadership influences teachers’ commitment and innovative practices: understanding the moderating role of trust in principal. Educ. Manage. Adm. Leadersh. 52, 455–474. doi: 10.1177/17411432221082803

[ref39] Kim J. (2024). Leading teachers’ perspective on teacher-AI collaboration in education. Educ. Inf. Technol. 29, 8693–8724. doi: 10.1007/s10639-023-12109-5

[ref40] Kirkpatrick S. A. Locke E. A. (1996). Direct and indirect effects of three core charismatic leadership components on performance and attitudes. J. Appl. Psychol. 81, 36–51. doi: 10.1037/0021-9010.81.1.36

[ref41] Leithwood K. Aitken R. Jantzi D. (2006). Making schools smarter: Leading with evidence. (3rd ed.). Thousand Oaks, CA: Corwin Press.

[ref42] Lewis A. Clark J. (2020). Dreams within a dream: multiple visions and organizational structure. J. Organ. Behav. 41, 50–76. doi: 10.1002/job.2419

[ref43] Li M. Liu F. Yang C. (2024). Teachers’ emotional intelligence and organizational commitment: a moderated mediation model of teachers’ psychological well-being and principal transformational leadership. Behav. Sci. 14:345. doi: 10.3390/bs14040345, 38667141 PMC11048059

[ref44] Li C. P. Shi K. (2005). The structure and measurement of transformational leadership. Acta Psychol. Sin. 37, 803–811.

[ref45] Li H. Zhao T. Li C. Pang X. (2023). Linking visionary leadership with employee creativity: perceived organizational support as a mediator. Soc. Behav. Pers. 51, 1–8. doi: 10.2224/sbp.12098

[ref46] Li L. Zhu H. Li H. (2022). School leadership enhances secondary students’ achievement in rural China through teacher commitment and collaborative culture. Front. Psychol. 13:894688. doi: 10.3389/fpsyg.2022.894688, 35677144 PMC9168753

[ref47] Liu S. Hallinger P. (2021). Unpacking the effects of culture on school leadership and teacher learning in China. Educ. Manag. Adm. Leadersh. 49, 214–233. doi: 10.1177/1741143219896042

[ref48] Liu S. Hallinger P. Feng D. (2016). Supporting the professional learning of teachers in China: does principal leadership make a difference? Teach. Teach. Educ. 59, 79–91. doi: 10.1016/j.tate.2016.05.023

[ref49] Liu Q. Sun Y. (2025). The impact of collaborative atmosphere on innovative work behavior of college teachers, North China. Front. Psychol. 15:1497503. doi: 10.3389/fpsyg.2024.1497503, 39877224 PMC11772426

[ref51] Liu S. Yin H. Wang Y. Lu J. (2024). Teacher innovation: conceptualizations, methodologies, and theoretical framework. Teach. Teach. Educ. 145:104611. doi: 10.1016/j.tate.2024.104611

[ref52] Lu C. Xu Z. Tian Q. (2025). Teachers’ well-being and innovative work behavior: a moderated mediation model of perceived insider status and principal authentic leadership. Behav. Sci. 15:1419. doi: 10.3390/bs15101419, 41153209 PMC12561759

[ref53] Ma X. Marion R. (2024). How does leadership affect teacher collaboration? Evidence from teachers in US schools. Sch. Eff. Sch. Improv. 35, 116–141. doi: 10.1080/09243453.2024.2330533

[ref54] Makhrus M. Sunardi O. Retnowati R. (2022). Increasing teachers’ creativity through the development of organizational culture, empowerment, and visionary leadership of school principals. Int. J. Soc. Manag. Stud. 3, 20–33.

[ref55] Mascareño J. Rietzschel E. Wisse B. (2020). Envisioning innovation: does visionary leadership engender team innovative performance through goal alignment? Creat. Innov. Manag. 29, 33–48. doi: 10.1111/caim.12341

[ref56] Maun D. Chand V. S. Shukla K. D. (2023). Influence of teacher innovative behaviour on students’ academic self-efficacy and intrinsic goal orientation. Educ. Psychol. 43, 679–697. doi: 10.1080/01443410.2023.2241682

[ref57] Meyer A. Hartung-Beck V. Gronostaj A. Krüger S. Richter D. (2023). How can principal leadership practices promote teacher collaboration and organizational change? A longitudinal multiple case study of three school improvement initiatives. J. Educ. Change 24, 425–455. doi: 10.1007/s10833-022-09451-9

[ref58] Murphy J. (2013). The architecture of school improvement. J. Educ. Adm. 51, 252–263. doi: 10.1108/09578231311311465

[ref59] Ozturk M. Wigelsworth M. Squires G. (2024). A conceptual model for teacher wellbeing: towards a holistic understanding. Cogent Educ. 11:2396156. doi: 10.1080/2331186x.2024.2396156

[ref60] Pan H. L. Lin Y. C. Chung C. H. (2024). Teacher collaboration, school innovativeness and innovative teaching in Taiwan: evidence from TALIS. Int. J. Educ. Res. 127:102383. doi: 10.1016/j.ijer.2024.102383

[ref61] Parker P. D. Martin A. J. Colmar S. Liem G. A. (2012). Teachers’ workplace well-being: exploring a process model of goal orientation, coping behavior, engagement, and burnout. Teach. Teach. Educ. 28, 503–513. doi: 10.1016/j.tate.2012.01.001

[ref62] Podsakoff P. M. MacKenzie S. B. Lee J. Y. Podsakoff N. P. (2003). Common method biases in behavioral research: a critical review of the literature and recommended remedies. J. Appl. Psychol. 88, 879–903. doi: 10.1037/0021-9010.88.5.879, 14516251

[ref63] Polatcan M. Özkan P. Bellibaş M. Ş. (2024). Cultivating teacher innovativeness through transformational leadership and teacher agency in schools: the moderating role of teacher trust. J. Prof. Cap. Community 9, 227–242. doi: 10.1108/jpcc-01-2024-0008

[ref64] Pozo-Rico T. Poveda R. Gutiérrez-Fresneda R. Castejón J. L. Gilar-Corbi R. (2023). Revamping teacher training for challenging times: teachers’ well-being, resilience, emotional intelligence, and innovative methodologies as key teaching competencies. Psychol. Res. Behav. Manag. 16, 1–18. doi: 10.2147/PRBM.S382572, 36636290 PMC9830420

[ref65] Preacher K. J. Rucker D. D. Hayes A. F. (2007). Addressing moderated mediation hypotheses: theory, methods, and prescriptions. Multivar. Behav. Res. 42, 185–227. doi: 10.1080/00273170701341316, 26821081

[ref66] Qi S. Derakhshan A. (2025). Positive emotions fuel creativity: exploring the role of passion and enjoyment in Chinese EFL teachers’ creativity in light of the investment theory of creativity. Appl. Linguist. Rev. 16:9. doi: 10.1515/applirev-2024-0109

[ref67] Qin S. Jia S. Su S. (2025). How does teacher collaboration impact teachers’ innovation ability? The chain mediation of teaching motivation and teaching efficacy. Humanit. Soc. Sci. Commun. 12, 1–12. doi: 10.1057/s41599-025-04965-y

[ref68] Rahimi H. Hejazi S. Y. Lou N. M. Heidarzadeh M. (2024). Are teachers with better quality of work life more innovative? The mediating roles of psychological empowerment and teaching mindsets. Acta Psychol. 247:104315. doi: 10.1016/j.actpsy.2024.104315 38749273

[ref69] Shamir B. House R. J. Arthur M. B. (1993). The motivational effects of charismatic leadership: a self-concept based theory. Organ. Sci. 4, 577–594. doi: 10.1287/orsc.4.4.577

[ref70] Song S. Gao L. Shi Y. Yu H. Wang W. (2025). Beacons of vision navigate career development: how and when does visionary leadership lead to followers’ career prospect? Leadersh. Organ. Dev. J. 46, 1100–1127. doi: 10.1108/lodj-10-2024-0641

[ref71] Stam D. Lord R. G. Knippenberg D. V. Wisse B. (2014). An image of who we might become: vision communication, possible selves, and vision pursuit. Organ. Sci. 25, 1172–1194. doi: 10.1287/orsc.2013.0891

[ref72] Starratt R. J. (1995). Leaders with Vision: the Quest for School Renewal. Thousand Oaks, CA: Corwin Press.

[ref74] Sun X. Liao W. (2025). “Lying flat” not involuting: an ethnographic case study on teacher disappointment in Chinese rural context. Teach. Teach. Educ. 155:104918. doi: 10.1016/j.tate.2024.104918

[ref75] Sun Y. Wang B. Ruan L. Liu X. Zhen R. (2025). Latent patterns and influencing factors of job burnout among primary and secondary school teachers in China. Teach. Teach. Educ. 159:104982. doi: 10.1016/j.tate.2025.104982

[ref76] Suyudi M. Rahmatullah A. S. Rachmawati Y. Hariyati N. (2022). The effect of instructional leadership and creative teaching on student actualization: student satisfaction as a mediator variable. Int. J. Instr. 15, 113–134. doi: 10.29333/iji.2022.1517a

[ref77] Teng Y. Pu R. Hao Y. (2024). How can an innovative teaching force be developed? The empowering role of distributed leadership. Think. Skills Creat. 51:101464. doi: 10.1016/j.tsc.2024.101464

[ref78] Thurlings M. Evers A. T. Vermeulen M. (2015). Toward a model of explaining teachers’ innovative behavior: a literature review. Rev. Educ. Res. 85, 430–471. doi: 10.3102/0034654314557949

[ref79] Van der Voet J. Steijn B. (2021). Team innovation through collaboration: how visionary leadership spurs innovation via team cohesion. Public Manag. Rev. 23, 1275–1294. doi: 10.1080/14719037.2020.1743344

[ref80] Van Knippenberg D. Sitkin S. B. (2013). A critical assessment of charismatic—transformational leadership research: back to the drawing board? Acad. Manage. Ann. 7, 1–60. doi: 10.5465/19416520.2013.759433

[ref81] Van Knippenberg D. Stam D. (2014). “Visionary leadership” in The Oxford handbook of leadership and organizations. ed. Day D. (Oxford: Oxford University Press), 241–259.

[ref82] Vangrieken K. Dochy F. Raes E. Kyndt E. (2015). Teacher collaboration: a systematic review. Educ. Res. Rev. 15, 17–40. doi: 10.1016/j.edurev.2015.04.002

[ref83] Vermeulen M. Kreijns K. Evers A. T. (2022). Transformational leadership, leader–member exchange and school learning climate: impact on teachers’ innovative behaviour in the Netherlands. Educ. Manage. Adm. Leadersh. 50, 491–510. doi: 10.1177/1741143220932582

[ref84] Vescio V. Ross D. Adams A. (2008). A review of research on the impact of professional learning communities on teaching practice and student learning. Teach. Teach. Educ. 24, 80–91. doi: 10.1016/j.tate.2007.01.004

[ref85] Wang T. (2018). “School leadership and professional learning community: case study of two senior high schools in Northeast China” in Global perspectives on developing professional learning communities. eds. Pang N. S. K. Wang T. (London: Routledge), 10–24.

[ref86] Wen Z. Hau K. T. Herbert W. M. (2004). Structural equation model testing: cutoff criteria for goodness of fit indices and chi-square test. Acta Psychol. Sin. 36:186.

[ref88] Wong J. L. (2010). What makes a professional learning community possible? A case study of a mathematics department in a junior secondary school of China. Asia Pac. Educ. Rev. 11, 131–139. doi: 10.1007/s12564-010-9080-6

[ref89] Xia W. Fan Y. Zhang X. Yang J. (2025). School organisational climate and job satisfaction among special education teachers in China: the roles of professional well-being and emotional exhaustion. Int. J. Disabil. Dev. Educ. 2025, 1–20. doi: 10.1080/1034912X.2025.2528210

[ref90] Xiang B. Xin M. Fan X. Xin Z. (2024). How does career calling influence teacher innovation? The chain mediation roles of organizational identification and work engagement. Psychol. Sch. 61, 4672–4687. doi: 10.1002/pits.23302

[ref91] Xu Z. Pang N. S. K. (2024). Promoting teachers’ organizational commitment: the effects of authentic leadership, teachers’ well-being and social–emotional competence. Behav. Sci. 14:862. doi: 10.3390/bs14100862, 39457734 PMC11505086

[ref92] Yang X. Shen J. Cropley D. H. Zheng Y. (2025). A systematic review of factors influencing K-12 teachers’ creative teaching across different forms: an ecological perspective. Think. Skills Creat. 57:101869. doi: 10.1016/j.tsc.2025.101869

[ref93] Zhai Y. Xiao W. Sun C. Sun B. Yue G. (2025). Professional identity makes more work well-being among in-service teachers: mediating roles of job crafting and work engagement. Psychol. Rep. 128, 2983–3000. doi: 10.1177/00332941231189217, 37535321

[ref94] Zhang S. Bowers A. J. Mao Y. (2021). Authentic leadership and teachers’ voice behaviour: the mediating role of psychological empowerment and moderating role of interpersonal trust. Educ. Manag. Adm. Leadersh. 49, 768–785. doi: 10.1177/1741143220915925

[ref95] Zhang W. Bowers A. J. Pang S. (2025). Promoting teacher knowledge sharing in China: the fostering roles of authentic leadership, reciprocity norms, and organizational trust. Educ. Manag. Adm. Leadersh. 53, 789–806. doi: 10.1177/17411432231199573

[ref96] Zhang L. Chen J. Li X. Zhan Y. (2024). A scope review of the teacher well-being research between 1968 and 2021. Asia Pac. Educ. Res. 33, 171–186. doi: 10.1007/s40299-023-00717-1

[ref97] Zheng X. Yin H. Liu Y. (2020). Are professional learning communities beneficial for teachers? A multilevel analysis of teacher self-efficacy and commitment in China. Sch. Eff. Sch. Improv. 32, 197–217. doi: 10.1080/09243453.2020.1808484

[ref98] Zheng X. Zhu W. Zhao H. Zhang C. H. I. (2015). Employee well-being in organizations: theoretical model, scale development, and cross-cultural validation. J. Organ. Behav. 36, 621–644. doi: 10.1002/job.1990

[ref99] Zhu C. Devos G. Li Y. (2011). Teacher perceptions of school culture and their organizational commitment and well-being in a Chinese school. Asia Pac. Educ. Rev. 12, 319–328. doi: 10.1007/s12564-011-9146-0

[ref100] Zhu C. Engels N. (2014). Organizational culture and instructional innovations in higher education: perceptions and reactions of teachers and students. Educ. Manag. Adm. Leadersh. 42, 136–158. doi: 10.1177/1741143213499253

[ref101] Zhu J. Yao J. Zhang L. (2019). Linking empowering leadership to innovative behavior in professional learning communities: the role of psychological empowerment and team psychological safety. Asia Pac. Educ. Rev. 20, 657–671. doi: 10.1007/s12564-019-09584-2

